# Case report of increased left ventricular end-diastolic pressure with pulsatile left ventricular assist device

**DOI:** 10.1093/ehjcr/ytae291

**Published:** 2024-06-24

**Authors:** Mauricio Felippi de Sá Marchi, Sarah Verhemel, Rutger-Jan Nuis, Nicolas M Van Mieghem

**Affiliations:** Department of Cardiology, Thoraxcenter, Erasmus University Medical Center, Rotterdam, The Netherlands; Department of Interventional Cardiology, Heart Institute (InCor), Hospital das Clínicas da Faculdade de Medicina da Universidade de São Paulo, São Paulo, Brazil; Department of Cardiology, Thoraxcenter, Erasmus University Medical Center, Rotterdam, The Netherlands; Department of Cardiology, Thoraxcenter, Erasmus University Medical Center, Rotterdam, The Netherlands; Department of Cardiology, Thoraxcenter, Erasmus University Medical Center, Rotterdam, The Netherlands

**Keywords:** Circulatory support, TAVI, High-risk PCI, Left ventricular assist device, Case report

## Abstract

**Background:**

Left ventricular assist devices (LVADs) are increasingly utilized in cardiogenic shock and high-risk percutaneous coronary interventions (PCIs). These devices aspirate and expel blood from the left ventricle (LV) into the aorta, consequently reducing left ventricular end-diastolic pressure (LVEDP). We report a case of unexpected LVEDP rise under LV-to-aorta LVAD in the context of transcatheter aortic valve implantation (TAVI) and concomitant multi-vessel PCI.

**Case summary:**

A patient with acute heart failure, severely depressed systolic LV function, severe aortic stenosis, and multi-vessel coronary artery disease underwent TAVI and concomitant PCI under pulsatile LVAD. Notably, the patient experienced unexpected shortness of breath and elevated LVEDP while under LVAD, which normalized immediately upon LVAD removal.

**Discussion:**

Pulsatile LVAD enhances cardiac output by providing pulsatile support through a percutaneous bi-directional flow catheter. Despite expectations of reduced LVEDP and improved myocardial oxygen supply under LVAD support, we observed high LVEDP and clinical complaints of shortness of breath following TAVI and multi-vessel PCI. This case illustrates that an LVAD across the aortic valve may immobilize aortic leaflets and generate acute aortic regurgitation.

**Learning points**

- Left ventricular end-diastolic pressure may rise during percutaneous cardiac interventions.
- The use of a pulsatile left ventricular assist device may inadvertently increase left ventricular end-diastolic pressure by inducing acute aortic regurgitation secondary to immobilization of aortic leaflets.

## Introduction

Percutaneous left ventricular assist devices (LVADs) are occasionally used for mechanical circulatory support during cardiogenic shock or high-risk percutaneous coronary interventions.^1^ Since its conception, LVAD has grown from a rescue strategy for cardiogenic shock patients to a bridging tool in various cardiovascular procedures.^1^ Current AHA/ACC guidelines suggest elective insertion of LVAD to facilitate percutaneous coronary intervention (PCI) in selected high-risk patients (class IIb, level of evidence B).^1,2^

Percutaneous LVADs are introduced across the aortic valve to aspirate and expel blood from the LV into the ascending aorta, which typically unloads the LV and reduces the left ventricular end-diastolic pressure (LVEDP).^1^ In this report, we describe a case in which LVEDP increased during LVAD support and immediately normalized upon device removal.

## Summary figure

**Figure:**
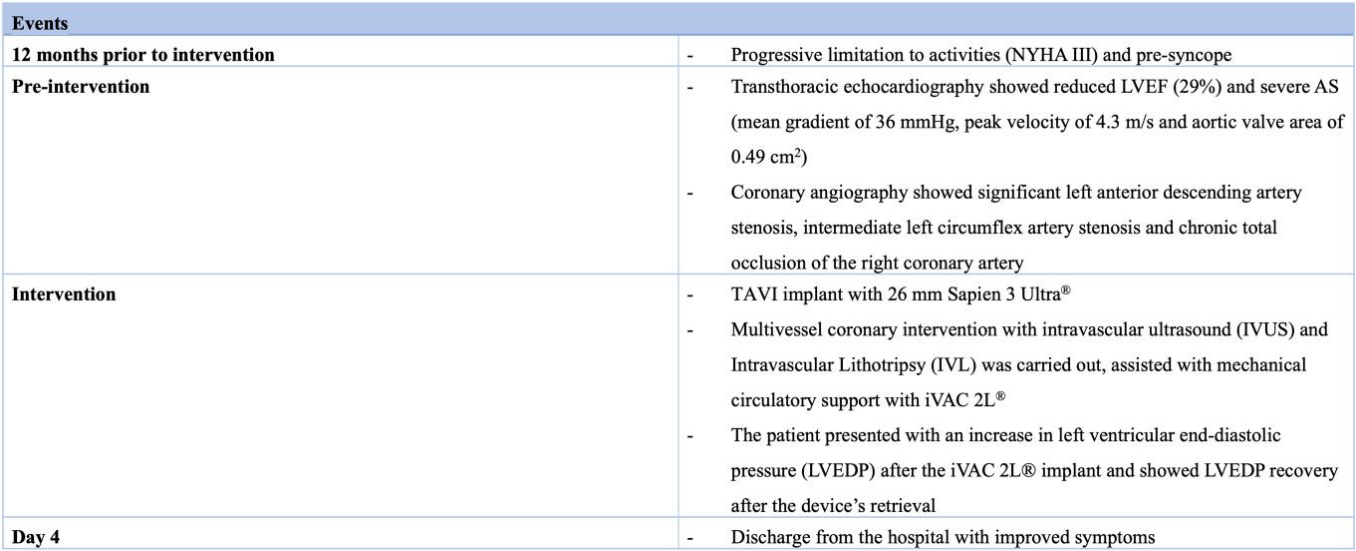


## Case presentation

**Figure 1:**
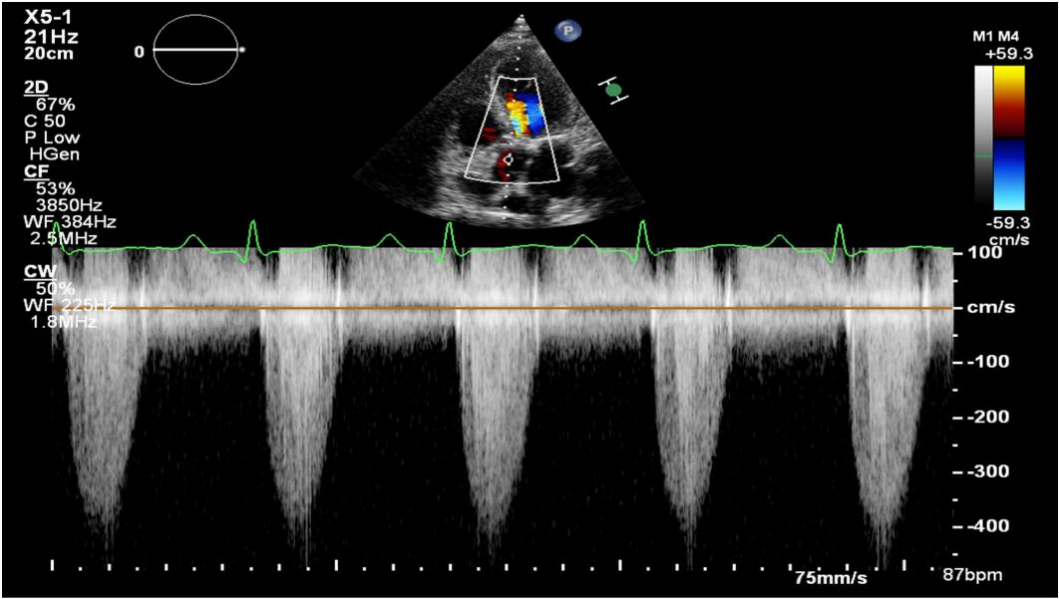
Continuous Doppler on native aortic valve. Continuous Doppler shows an aortic valve peak velocity of 4.31 m/s.

**Figure 2:**
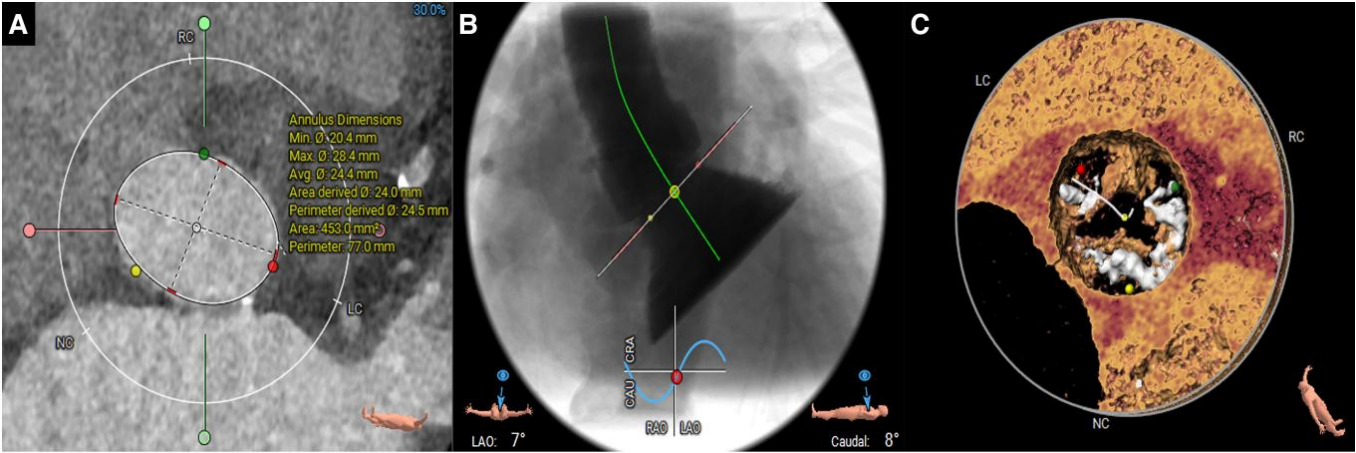
Multidetector computed tomography images of the native aortic valve. (*A*) Measurement of aortic annulus for cross-sectional area and perimeter in end systole. (*B*) Three-cusp coplanar view. (*C*) Three-dimensional reconstructions of valve.

**Figure 3:**
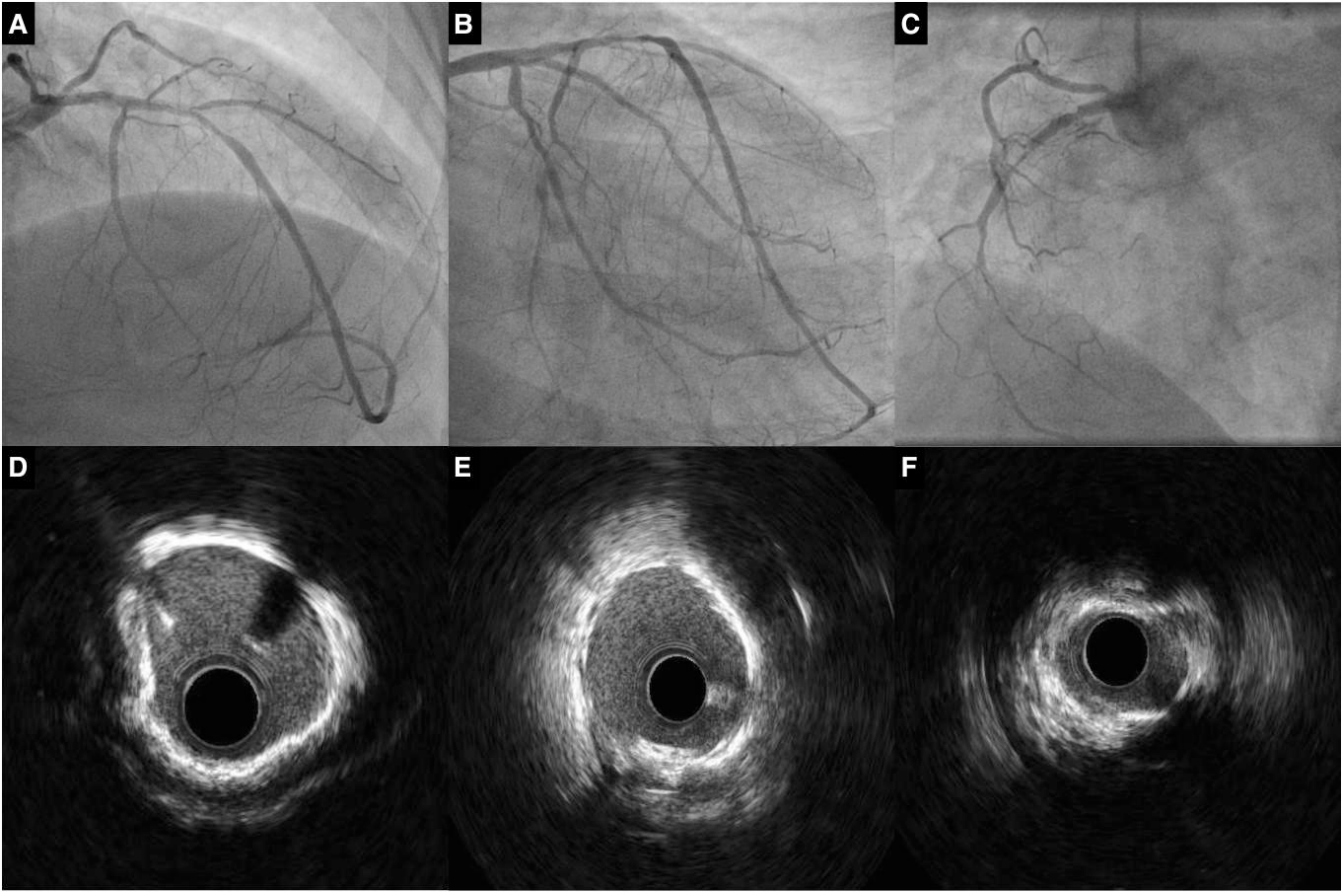
Coronary angiographies and intravascular ultrasound (IVUS) evaluation. (*A*) Cranial angiography showcasing left anterior descending (LAD) severe stenosis in the proximal and medium segment of the vessel. (*B*) Right caudal angiography showing left circumflex (LCx) severe stenosis in the proximal segment of the vessel. (*C*) Left oblique angiography displaying diffuse severe right coronary artery (RCA) stenosis. (*D*) IVUS of the LAD presenting a 360° calcification arch. (*E*) IVUS of the LCx revealing a 180° calcification arch. (*F*) IVUS of the RCA showing diffuse calcification and vessel sub-occlusion.

A 57-year-old man with a history of hypercholesterolaemia, type 2 non-insulin dependent diabetes mellitus, and obstructive sleep apnoea was admitted with dyspnoea and peripheral oedema, secondary to decompensated heart failure (NYHA class IV). On physical examination, auscultation revealed a parasternal holosystolic murmur over the right 2nd intercostal space and bilateral pulmonary crepitations. Laboratory tests showed a serum creatinine of 105 mmol/L, GFR 68 mL/min, NT-pro-BNP value of 664 pmol/L, and maximum high-sensitive troponin 345 ng/L. Transthoracic echocardiography (TTE) revealed reduced LVEF (29%) and severe aortic stenosis (AS), with a mean gradient of 36 mmHg, peak velocity of 4.3 m/s, and aortic valve area of 0.49 cm2 (*Figure 1*). Multi-slice computed tomography exhibited an Agatston score of 3194 AU and an annulus area of 453 mm^2^ (*Figure 2*). Coronary angiography showed significant left anterior descending artery (LAD) stenosis, intermediate left circumflex artery (LCx) stenosis, and chronic total occlusion of the right coronary artery (RCA) (*Figure 3*).

The multidisciplinary heart team deemed the patient at high operative risk because of the decompensated AS, severely depressed LV function, and multi-vessel coronary disease, calculated STS PROM 4.2% and reached consensus for transcatheter aortic valve implantation (TAVI) with concomitant PCI under pulsatile LVAD. This decision was in accordance with the joint Dutch cardiology society (NVVC) and Dutch cardiothoracic surgeons society (NVT) consensus statement for the indication of TAVI in 2020.^3^

The procedure was performed under local anaesthesia and filter based cerebral embolic protection (Sentinel®, Boston Scientific, Marlborough, USA). A 14 F re-collapsible sheath was introduced in the right common femoral artery and a 6 F sheath in the ipsilateral superficial femoral artery. Invasive pressures were measured using a pig-tail catheter positioned at the LV apex and the aorta. Initial LVEDP was 43 mmHg, and the transvalvular mean aortic pressure gradient was 67 mmHg. A 0.035″ pre-shaped stiff wire (Safari®, Boston Scientific, Marlborough, USA) was advanced and positioned in the LV. Aortic valve balloon valvuloplasty with a 22 mm balloon and TAVI with a 26 mm Sapien 3 Ultra® (Edwards Lifesciences, Irvine, USA) was performed under rapid pacing on the 0.035″ pre-shaped stiff wire (Safari®, Boston Scientific, Marlborough, USA) in the LV. The valve was subsequently post-dilated with a 24 mm balloon due to angiographic evidence of aortic regurgitation. After TAVI LVEDP dropped to 24 mmHg and final aortography showed no signs of residual aortic insufficiency.

**Figure 4:**
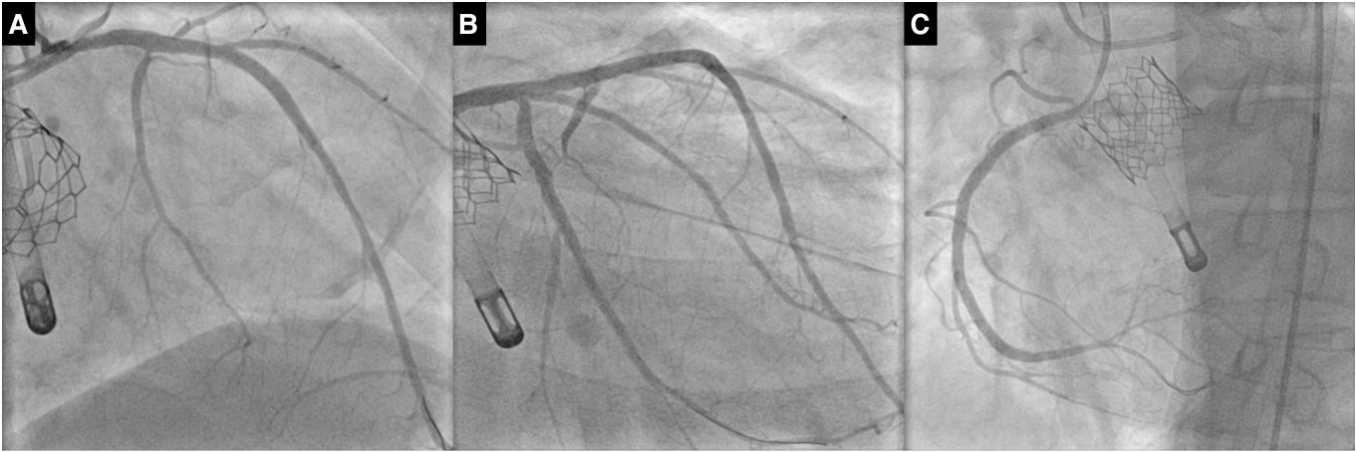
Final coronary angiographies. (*A*) LAD angiography. (*B*) LCx angiography. (*C*) RCA angiography.

**Figure 5:**
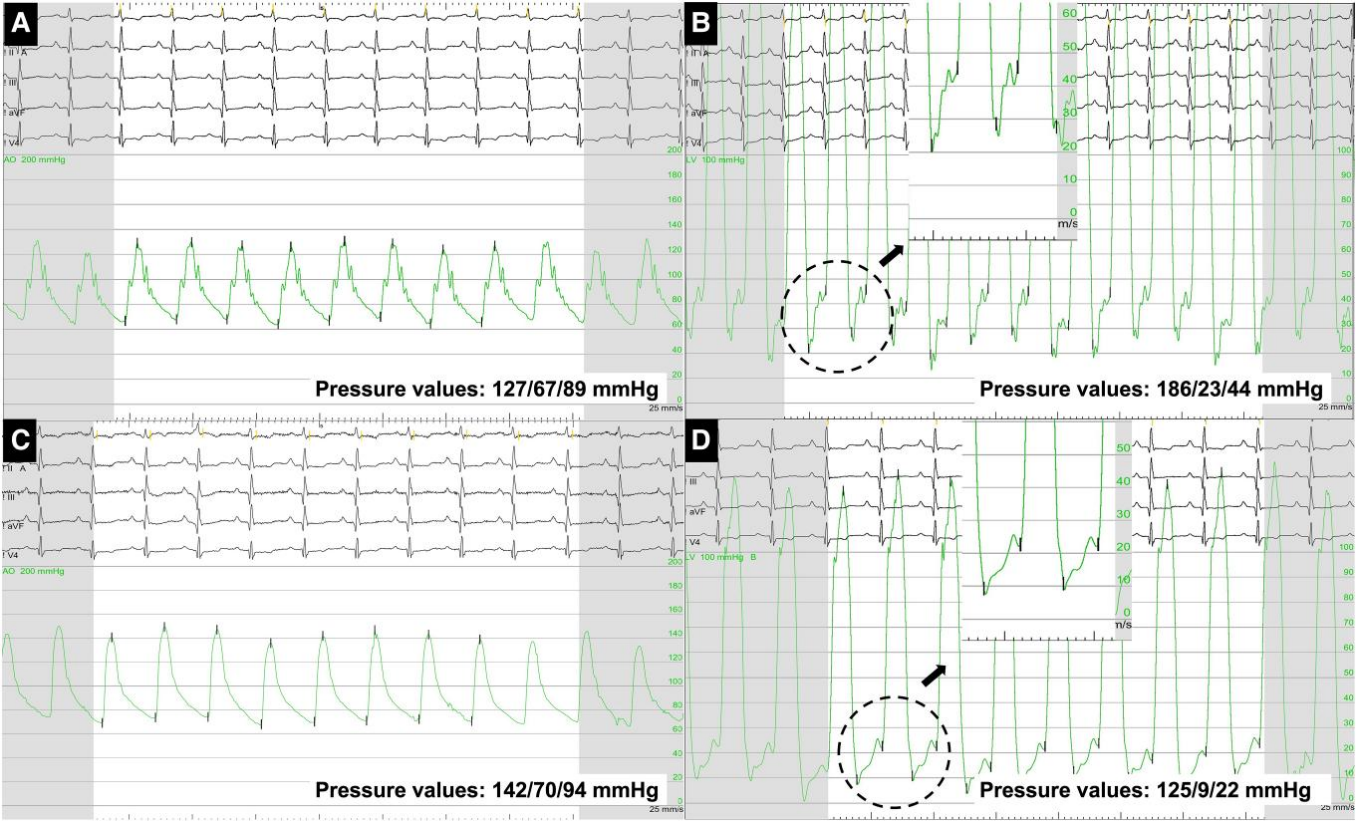
Aortic pressure and left ventricular end-diastolic pressure (LVEDP) curves. Aortic pressure (*A*) and left ventricular end-diastolic pressure (*B*) curves with iVAC 2L. Aortic pressure (*C*) and left ventricular end-diastolic pressure (*D*) curves after the device retrieval.

After TAVI, a 17 F pulsatile LVAD device (iVAC 2L®, PulseCath BV, Amsterdam, The Netherlands) was introduced over the 0.035″ LV wire to provide support for PCI. The patient noticed respiratory discomfort upon iVAC2L insertion. Intravascular ultrasound (IVUS)-guided complete revascularization was performed including calcium modification with a 3.0 mm Shockwave balloon® (Shockwave Medical, Santa Clara, USA) and a 3.5 × 40 mm drug-eluting stent (DES) in the LAD, a 2.5 × 26 mm DES in the LCx, and successful recanalization of the RCA with four (4.0 × 9 mm, 3.5 × 40 mm, 3.5 × 40 mm, and 2.5 × 15 mm) DES (*Figure 4*). Left ventricular end-diastolic pressure after multi-vessel PCI completion under LVAD support was 44 mmHg. After its removal, LVEDP immediately dropped to 22 mmHg (*Figure 5* and Supplementary material online, *Video S1*). Large bore common femoral arteriotomy closure was successfully achieved with two Perclose ProStyle (Abbott, Abbott Park, USA) devices and one 8 F Angio-Seal (Terumo Interventional Systems, Tokyo, Japan). After confirmation of patent haemostasis by angiography through the SFA sheath, superficial femoral arteriotomy was closed with one perclose prostyle device.

Pre-discharge TTE displayed an overall good bioprosthetic valve performance, with aortic mean gradient of 14 mmHg, no paravalvular leak, and 35% EF.

Patient experienced a fast recovery after the procedure. The LVEF improved to 35%, the aortic valve area was 1.7 cm2, and the patient was discharged on Day 2 after the procedure.

## Discussion

Multiple percutaneous LVADs with variable profile and mode of operations are available.^1^ Patients undergoing complex PCI may be at risk for haemodynamic compromise but,^2^ current evidence does not support the routine utilization of LVAD devices to mitigate cardiovascular events during complex PCI. Clinical judgement is advised on a case-by-case basis (class IIb, level of evidence B).^2^

In our case, the patient was deemed at high surgical risk and also at risk for haemodynamic compromise during PCI because of the decompensated severe AS in combination with low LVEF and multi-vessel complex coronary disease that may require advanced lesion preparation techniques. We opted for a percutaneous approach with TAVI and PCI under LVAD support with the iVAC 2L (PulseCath BV, Amsterdam, The Netherlands), a percutaneous 17 Fr bi-directional flow catheter that generates up to 2.0 L/min of pulsatile support on top of the existing cardiac output.^4^ This catheter is inserted through the common femoral artery and advanced retrogradely across the aortic valve into the LV. The LVAD distal tip should be positioned midway within the left ventricular cavity and the two-way valve containing outlet in the ascending aorta.^5^ After introduction, the catheter is connected to an extracorporeal membrane pump, which is driven by an intra-aortic balloon pump console. Using electrocardiographic triggering, blood is aspirated from the LV through the inlet tip during systole and ejected via the two-way valve into the ascending aorta during diastole.^5^ The resulting pulsatile support generates additional output with systolic unloading of the LV and diastolic counterpulsation.^4,5^ The pivotal valve function at the outflow port is synchronized with aortic valve closure and should not interfere with the aortic valve functioning.^4,5^

Unexpectedly, after TAVI and the introduction of the iVAC2L, the patient experienced immediate respiratory discomfort. After completion of the multi-vessel PCI, the LVEDP remained elevated despite LVAD support. Upon LVAD removal, LVEDP promptly decreased. Our primary hypothesis for this occurrence was the eccentric position of the LVAD across the aortic valve, potentially leading to partial immobilization or restricted mobility of one or more aortic leaflets, resulting in inadequate leaflet coaptation and severe valvular aortic regurgitation. In order to prevent this complication, physicians should be mindful of LVAD malpositioning across the aortic valve and have a low threshold for echocardiography in the context of unexplained haemodynamic compromise under mechanical circulatory support.

## Conclusion(s)

Left ventricular assist device can potentially cause malcoaptation of TAVI leaflets, leading to acute aortic regurgitation (AR) due to partial immobilization or restricted mobility of one or more aortic leaflets. This inadequate leaflet coaptation can result in severe valvular AR. Thus, it is essential to ensure precise device placement and promptly detect any aortic leaflet malcoaptation to prevent this rare occurrence.

## Supplementary Material

ytae291_Supplementary_Data

## Data Availability

The data underlying this article are available in the article and in its online Supplementary material.

## Lead author biography

Board-certified interventional cardiologist by the Brazilian Society of Interventional Cardiology and Hemodynamics. Interventional cardiology fellow at the Heart Institute (InCor—HCFMUSP), São Paulo, Brazil. Structural heart interventions fellow at the Heart Institute (InCor—HCFMUSP), São Paulo, Brazil. PhD candidate at the University of São Paulo (USP), Brazil. Interexchange PhD fellow at the Erasmus Medical Center (EMC), Rotterdam, The Netherlands.

## Supplementary material

**Consent:** The authors confirm that written consent for the submission and publication of this case, including images, has been obtained from the patient and his parents in line with COPE guidance.

**Funding:** M.F.d.S.M. is supported by a PhD Scholarship for International Research from ‘Conselho Nacional de Desenvolvimento Científico e Tecnológico-Brasil (CNPq)’, under grant: 88887.716769/2022-00. N.M.V.M. has received grant funding from or has contracts with Abbott, Boston Scientific, Biotronik, Edwards Lifesciences, Medtronic, PulseCath BV, Abiomed, and Daiichi Sankyo; consulting fees from JenaValve, Daiichi Sankyo, Abbott, Boston Scientific, and Medtronic; payments or honoraria for lectures, presentations, speaking, manuscripts, and educational events from Abiomed and Amgen; and support for attending meetings and or travel from JenaValve.
